# The factors influencing car use in a cycle-friendly city: the case of Cambridge

**DOI:** 10.1016/j.jtrangeo.2012.10.013

**Published:** 2013-04

**Authors:** Andrew Carse, Anna Goodman, Roger L. Mackett, Jenna Panter, David Ogilvie

**Affiliations:** a Centre for Transport Studies, University College London, Gower Street, London WC1E 6BT, UK; b Faculty of Epidemiology and Population Health, London School of Hygiene and Tropical Medicine, Keppel Street, London WC1E 7HT, UK; c Medical Research Council Epidemiology Unit and UKCRC Centre for Diet and Activity Research (CEDAR), Box 296, Institute of Public Health, Forvie Site, Robinson Way, Cambridge, UK

**Keywords:** Cars, Cycling, Travel behaviour, Multivariable regression

## Abstract

Encouraging people out of their cars and into other modes of transport, which has major advantages for health, the environment and urban development, has proved difficult. Greater understanding of the influences that lead people to use the car, particularly for shorter journeys, may help to achieve this. This paper examines the predictors of car use compared with the bicycle to explore how it may be possible to persuade more people to use the bicycle instead of the car. Multivariable logistic regression was used to examine the socio-demographic, transport and health-related correlates of mode choice for work, shopping and leisure trips in Cambridge, a city with high levels of cycling by UK standards. The key findings are that commuting distance and free workplace parking were strongly associated with use of the car for work trips, and car availability and lower levels of education were associated with car use for leisure, shopping and short-distanced commuting trips. The case of Cambridge shows that more policies could be adopted, particularly a reduction in free car parking, to increase cycling and reduce the use of the car, especially over short distances.

## Highlights

► Comparison of the use of the car and bicycle for work, shopping and leisure trips. ► Multivariate logistic regression examines correlates associated with modal choice. ► Distance and free workplace car parking are associated with car use for commuting. ► Car access and education effect car use on shopping, leisure and short work trips. ► Policies are available to encourage modal shift even in cities with high cycling.

## 1 Introduction

In transport research, considerable attention has been devoted to the question of how to get people out of their cars (Hensher, 1998; Stradling, 2003). It is, however, difficult to turn this aspiration into practice. A reduction in short trips by car is important for the future of cities (Monzon et al., 2011) and could also bring benefits for health, the environment and quality of life (Grabow et al., 2012; Mackett, 2003; Maibach et al., 2009). This paper examines the predictors of car use compared to bicycle use in a city with a traditional cycling culture (Aldred, 2010) in order to explore the possible implications for other areas. Attention is paid to the bicycle because it can provide a genuine sustainable alternative to the car for many trip purposes. For short trips there are really only three alternatives to the car in most areas; the bus, walking and cycling. While bus travel and walking provide alternatives in some settings, in others inadequate timetables and poor network coverage limit how effectively buses can compete with the car, and there is a limit to how far people can be expected to walk. In other parts of Europe, cycling accounts for a much higher modal share, up to 26% of all trips in the Netherlands and 16% in Denmark (Cycling Embassy of Denmark, 2010, Ministry of Transport Public works and Water Management, 2009). In the UK, however, as in all western countries, the car is the dominant mode of transport: data from the National Travel Survey (Department for Transport, 2010) shows that 63% of all trips are made by car compared to just 2% by bicycle. The car is the main mode for commuting and business (69%), shopping (64%) and leisure trips (69%), whereas for the bicycle the equivalent proportions are 3%, 1% and 2% respectively. It is not clear whether the bicycle can effectively compete with the car in the UK, given that cars have become an integral part of everyday life for many households (Katz, 1999). It was comparable in the 1950s however, with more traffic by vehicle for bicycles than cars in 1949 (Department for Transport, 2011b). Since then car use has continued to grow and cycling declined. The car has certain advantages over other modes in terms of speed, flexibility, safety and personal space. But car travel can have negative aspects for the user, such as being a very stressful experience (Novaco et al., 1990; Rasmussen et al., 2000), whilst cycling can be pleasant and exciting (Gatersleben and Uzzell, 2007). There are also health benefits of travelling by bicycle. Studies have shown that cycling can reduce the risk of cardiovascular disease and premature mortality (Andersen et al., 2000; Bauman and Rissel, 2009) and that the health benefits of a shift towards walking and cycling (sometimes known as active travel) are likely to strongly outweigh the harms (de Hartog et al., 2010). For car users to change their travel behaviour, however, a desire for change, clear benefits and the availability of a viable alternative are likely to be required (Stradling et al., 2000).

In the UK there has been an increased focus on cycling following a shift in policy direction dating from the White Paper ‘A New Deal for Transport’ (DETR, 1998). The government of the time subsequently introduced a long-term strategy to encourage people to use more sustainable modes of travel (Cairns et al., 2004). The first part of the strategy included the Cycling Demonstration Town programme that started in October 2005 and provided investment for six towns. Each town received funding that equated to £10 per head of population per year, sourced equally from central and local government (Sloman et al., 2009). The Department for Transport and the Department of Health followed this with a further £43 m invested in a second phase known as the Cycling City and Towns (CCTs) programme involving one city and 11 towns. The aim was to explore whether increased investment in cycling as part of a whole-town strategy could lead to a significant and sustained increase in the number of cyclists and the frequency of cycling (Department for Transport, 2011a). Evaluation of the Cycling Demonstration Towns reported an average 27% increase in cycling relative to levels in 2005 before the introduction of the programme (Sloman et al., 2009). The aim of this study is to identify which characteristics are significantly associated with the choice of the car versus the bicycle for work, shopping and leisure trips. The study takes place in one of the Cycling Towns, Cambridge. This is a location with a history of high levels of cycle use compared to other urban areas in the UK. Indeed, Cambridge has the UK’s highest modal share for cycling to work (25%), substantially higher than that for the locations with the next highest modal shares (Oxford, 14% and York, 14%) (ONS, 2001). Investigating why people continue to use the car in an area with a high prevalence of cycling may help inform strategies that could increase cycling in other towns and cities in order to bring about improvements for traffic congestion and public health. Kingham et al. (2001) have found that many factors are discouraging people to move out of their car and onto their bicycle, including distance, cycle infrastructure and because there is too much traffic on the roads. However in Cambridge, which has been described as a city representing a cycling culture, there are factors that encourage cycling, including having a favourable flat environment (of with parts of the city centre closed to motor traffic), a generally temperate climate (the region’s mean temperatures are higher than the UK average and has lower rainfall and wind levels (Met Office, 2012)), prominent cycling activism and extensive infrastructure (Aldred, 2010). Therefore if predictors of car use can be identified, these may help inform actions that could be introduced to increase cycling in other locations. The study also examines the specific correlates of modal choice for short work trips (those of less than 5 km) to examine whether there are policies that might help to promote modal shift for these trips (Mackett, 2001).

## 2 Methods

This analysis uses data collected as part of the Commuting and Health in Cambridge study, which is being conducted in Cambridge, UK and has been described in more detail elsewhere (Ogilvie et al., 2010). In summary, a questionnaire survey of working adults (aged 16 and over) was conducted between May and October 2009. Participants were recruited through workplaces in Cambridge to which they commuted from within an approximate radius of 30 km of the city centre. The questionnaire included a 1-day travel record of all trips made on the previous day (Panter et al., 2011). This had been used in a previous study in Glasgow (Ogilvie et al., 2008) and adapted from the UK National Travel Survey (Stratford et al., 2003). For each trip, respondents specified the purpose and elapsed time spent using each travel mode. The trips were classified using the National Travel Survey categories for trip purpose and main mode (Department for Transport, 2010). Of the eight trip purposes, work, shopping and leisure trips were used in the analysis because they were the most frequently reported categories. *Factors affecting modal choice* To characterise those who used the car (compared to the bicycle) for different trip purposes, three main groups of explanatory variables were considered: socio-demographic, transport and health-related indicators, all of which were taken from the relevant sections of the questionnaire (Panter et al., 2011). Socio-demographic indicators included sex, age, presence of children in the household, education, housing tenure and urban–rural status. Binary indicators were created for having children aged under 5 years or between 5 and 15 years, whether the participant’s home was rented or owned, and whether the participant lived in an urban or rural location. This last variable was determined according to the Urban and Rural Classification of the participants’ residential Census Output Area (Bibby and Shepherd, 2004). Age was categorised into five bands and education was classified into four groups of highest level of attainment – degree level, ‘A’ Level or equivalent, GCSE or equivalent and other. Transport indicators included having a driving licence, having access to cars and bicycles and the frequency of walking for pleasure. Binary indicators were produced for holding a driving licence and for bicycle access. Three categories were derived for the number of cars per adult in the household: none, less than one (which included households with one or more cars available but fewer cars than adults in the household) and one or more. Time spent walking for pleasure was included in order to identify any association between recreational walking and modal choice. This variable was derived from the total reported duration of walking for pleasure in the past week (in minutes) and categorised into four groups. Finally two additional transport variables were used in the analysis of work trips (but not shopping and leisure trips): parking provision at work (categorised as free parking, paid parking or no parking) and network distance from home to work, which was computed in a Geographical Information System (GIS) (ArcGIS 9.3) using home and work postcodes provided in the questionnaire and categorised as less than 3 km, 3–5 km, 5–10 km or greater than 10 km.

The health-related indicators were body mass index (BMI) and the physical and mental health summary scores of the SF-8 (Ware et al., 2001). BMI was calculated by dividing weight in kilograms by height in metres squared and categorised into one of three groups (World Health Organisation, 2000): underweight/normal weight, overweight and obese. The SF-8 physical (PCS-8) and mental (MCS-8) health summary scores provide a reliable measure of physical and mental health based on eight questions on general health, physical functioning, and limitations over the past 4 weeks due to physical health problems, bodily pain, energy, social functioning, mental health and emotional problems (Ware et al., 2001). Responses were given on Likert scales. PCS-8 and MCS-8 summary scores were then calculated using the method and coefficients given in the SF-8 manual (Ware et al., 2001).

Univariable and multivariable logistic regression analyses were conducted for the individual trip purposes to provide separate models for work, shopping and leisure trips. In all cases the outcome measure was modal choice (0 = bicycle and 1 = car). Trips made for other purposes, and trips made using a main mode other than the bicycle or the car, were excluded from analysis. Univariable associations were identified for each explanatory indicator to estimate the odds ratio (OR) for using a car instead of cycling for each trip purpose. As suggested by Hosmer and Lemeshow (2000), only variables for which a significance level of less than 0.25 was obtained in univariable analysis were included in the multivariable logistic regression models. Multivariable modelling began with the entry of socio-demographic variables, followed by transport variables and finally health variables. This sequential model building was designed to explore the relative importance of the three domains of explanatory variables and how these varied when variables from each domain were added. If a variable had a significance level greater than 0.05 in the socio-demographic (Model 1), transport (Model 2) or health (Model 3) models it was removed and only the significant variables were included in the tables and the final model (Model 4). Because the analysis was conducted at trip level there were often multiple trips by the same individual; robust standard errors were used to account for this clustering. All analysis was conducted in Stata version 10.0.

## 3 Results

Eleven hundred and sixty four completed questionnaires were returned, and in these a total of 4124 trips were recorded. Of these, 3784 trips were valid: trips were discounted if entries were incomplete or trips had more than one purpose of which the main purpose could not be deciphered. The majority of the 3784 trips were for work (1906, 50%); 609 (16%) were made for shopping and 636 (17%) for leisure. The remaining 17% of trips for all other purposes were not included. As the analysis is comparing the bicycle with the car, only trips made by these modes were used. Descriptive statistics for each of the trip purposes made using either the bicycle or the car as the main mode are provided in Table 1.

### 3.1 Work travel

A summary of the multivariable analysis of work trips is shown in Table 2. The most prominent initial finding was the extent to which the transport characteristics (Model 2 in Table 2) contributed to modal choice. Their coefficient of determination (pseudo *R*^2^) was 0.45, which indicates that 45% of variance in the choice of the car over the bicycle was explained by the transport predictors. It should be noted that the value of the pseudo *R*^2^ is not comparable to an *R*^2^ value obtained using ordinary least squares (OLS) regression, but it can still be used as a representation of how the model is performing and the relative influence of different groups of explanatory variables (Hensher et al., 2005). In this model, participants with more cars per adult in the household or commuting from greater distances were more likely to commute by car. Two other variables were significantly associated with car use: having free workplace parking, and walking 20–30 min per week for pleasure.

When socio-demographic, transport and health related characteristics were included in one model (Model 4 in Table 2), 52% of the variance in car travel was explained by the model, with the largest contribution coming from the transport characteristics. In this group the statistically significant variables were commuting distance, car ownership and free workplace parking. The other explanatory variables that were significant in the model were being female, the possession of only ‘A’ Level qualifications or equivalent, and a BMI in the obese range. Variables that were significant in domain-specific models and not in Model 4 included living in an owner-occupied property and living in a rural location (Model 1, Table 2) and PCS-8 and MCS-8 (Model 3, Table 2).

Because commuting distance showed one of the strongest associations with car commuting, the associations of the other socio-demographic, transport and health-related characteristics within shorter and longer trips were examined separately. In the model for short trips (defined as commuting trips made by participants with a computed home-to-work network distance of less than 5 km) fewer variables were significantly associated with modal choice (Table 3) and the pseudo *R*^2^ value decreased to 0.25. The transport characteristics (Model 2 in Table 3) still showed the strongest associations with modal choice, but less so than in the overall model. Cars per adult and workplace parking were statistically significant predictors, with the likelihood of car use being increased if there were one or more cars per adult per household and if participants reported having free car parking at work. In the socio-demographic model only the possession of ‘A’ Level qualifications was significant (Model 1 in Table 3) and only having a BMI classed as obese was significant in the health model (Model 3 in Table 3). This association with BMI did not persist in Model 4 after adjustment for socio-demographic and transport characteristics (Model 4 in Table 3). Instead, those with lower educational qualifications were more likely to report making work trips by car. The only two other significant predictors of modal choice in this model were the availability of one or more cars per adult in the household and free workplace parking.

### 3.2 Shopping travel

For shopping trips, the transport variables that were statistically significant were car ownership and walking for pleasure (Model 2 in Table 4). The more cars available, the more likely participants were to travel by car and if the participant walked for 20–30 min for pleasure per week, the likelihood of car travel was also increased. In the model including only socio-demographic characteristics (Model 1 in Table 4), age, housing tenure and education were associated with the likelihood of using the car for shopping trips. In the health model, no explanatory variables were significantly associated with modal choice.

When the three domains of explanatory variables were combined (Model 4) the variance in modal choice explained by the model increased to 36%. Age, education and car ownership were the main factors associated with modal choice for shopping. Participants aged 40–49 or 60 years and over, and those without a degree-level education, were more likely to travel by car. Once again, the higher the number of cars available per adult in the household, the more likely that the trip would be made by car. None of the health characteristics were associated with modal choice in this model, and only the effect of walking for pleasure for 20–30 min did not remain significant from Model 2 to Model 4.

### 3.3 Leisure travel

The analysis of leisure trips produced a similar result to that for the shopping trips. The variables studied contributed relatively little to explaining the choice of the car over the bicycle, with similar pseudo *R*^2^ figures for the socio-demographic and transport characteristics. When all the groups of explanatory variables were included, the pseudo *R*^2^ value increased to 27% (Model 4 in Table 5) in line with findings for the other trip purposes. Education, car ownership and BMI were predictors of modal choice. Lower levels of educational attainment, more cars per adult in the household and being classed as obese were all associated with travelling by car instead of cycling. For the socio-demographic model (Model 1 in Table 5), the predictors of car use that did not remain in Model 4 were being female and living in a rural location.

## 4 Discussion

The objective of this paper was to identify predictors that are associated with the choice of the car over the bicycle for different trip purposes. Using the case study of Cambridge as a city with high cycle use, the socio-demographic, transport and health-related characteristics of work, shopping and leisure trips were examined. Key findings were that (i) commuting distance and workplace car parking availability were strongly associated with using the car to travel to work; (ii) similar socio-demographic, transport and health-related characteristics were associated with car commuting for both long and short trips to work; and (iii) access to a car and lower levels of education were both associated with an increased likelihood of using the car for shopping, leisure and short-distance commuting trips.

Commuting distance was strongly associated with the choice between the bicycle and the car for work trips. The importance of commuting distance probably reflects the fact that people living closer to work, shops and leisure facilities are more likely to be able to cycle, whereas those driving may tend to do so because they live further from these key destinations. More compact urban form is difficult to achieve in established towns and cities, but the type and location of new developments should be considered in terms of the impact they may have on travel behaviour. In the Netherlands, it has been shown that strategic national spatial planning has been effective in retaining high shares of cycling and walking in the large and medium-sized cities (Schwanen et al., 2004).

In this study, car travel was not associated with higher socio-economic classification. Whereas previous research has shown that travelling by car is associated with the highest level of education (Schwanen et al., 2002), here an inverse relationship was found in that the lower the level of education, the more likely that travel for work, shopping and leisure purposes was made by car rather than by bicycle. Travel to work data using the National Statistics Socio-Economic Classification (NS-SEC) for Cambridge shows there are a greater proportion of cyclists in higher occupational categories than in the lower (30% compared with 23%) (ONS, 2001). There is also a larger number driving with a lower NS-SEC (41%) compared to those in a higher NS-SEC (34%). In the higher occupational categories, having a degree is by far the most frequent level of qualification whereas in the lowest NS-SEC categories, having no qualifications is the most commonly reported level of educational attainment. As has been previously argued, this is likely to reflect the fact that in this sample, not owning a car is generally not a marker of deprivation. Instead, it typically reflects those living close enough to the city centre do not to need a car which, given high housing costs in central Cambridge, could be a marker for greater affluence. Both in this sample and in Cambridge as a whole, levels of education are higher among individuals living closer to the city centre (Goodman et al., 2012).

Free workplace car parking was strongly associated with car use for commuting (including short trips) and could be considered a deterrent to cycling. In the US it has been suggested that removing free parking could reduce car travel to work by up to 81% (Willson and Shoup, 1990). One possible intervention strategy would be to implement a workplace parking levy such as that introduced in Nottingham (Nottingham City Council, 2011). Employers that provide 11 or more workplace parking spaces are now required to pay £279 per year per place. The revenue generated is to be used to fund improvements on public transport.

While car ownership was, unsurprisingly, strongly associated with not cycling, it is has been shown that car ownership was not necessarily incompatible with cycle use. Almost 80% of cycle trips to and from work in this sample were made by cyclists from car-owning households, and in almost 25% of cases there were one or more cars available per adult in the household. This may reflect what has been described as Cambridge’s ‘cycling citizenship’ (Aldred, 2010), whereby the independence or freedom embodied in cycling has become part of daily life. It also suggests that a shift towards a cycling culture in other towns and cities (such as those targeted in the CCT programme) may be achievable even in a context of relatively high car ownership and use.

With these conclusions in mind there are a number of study limitations to consider. The first is that the sample was not representative of the UK or even of all commuters in Cambridge. In this sample, 53.3% of trips to work are by bicycle compared to 24.9% in Cambridge according to the UK census (ONS, 2001). Table 1 shows that bicycle use is even more prevalent for short trips. The second relates to the method of measuring travel behaviour used in the analysis, which was based on a 1-day travel record and (for this analysis) limited to two specific main modes. While other modes of travel (such as walking or bus use) could have been included in the analysis, Cambridge presented a particular opportunity to make a direct comparison between the car and the bicycle. When longitudinal data from this study are available we will be able to analyse the determinants of travel behaviour (and change in travel behaviour) more effectively rather than relying on a cross-sectional analysis of the correlates of modal choice. This will also be enhanced by evaluation of travel diary data to explore travel patterns throughout the week and within households. A further step in this research would be to monitor the growth of cycling in other areas, particularly the CCTs, in order to understand why people continue to use the car, especially for shorter trips, particularly after infrastructure and policy measures to support cycling have been implemented effectively. Even with these limitations there are policy recommendations that can be made. The limitations do not affect the influence that potential changes could have on travel behaviour. When the additional data becomes available a stronger case will be made for how people could be encouraged out of the car.

Cycling accounts for the minority of modal share in almost all urban locations of the UK, where the car is dominant. The case of Cambridge shows that more could be done to increase cycling and reduce the use of the car, especially over short distances. Even when a ‘cycling culture’ is not apparent, more policies could be introduced that might lead to an increase in cycling, one obvious example being a reduction in free car parking. CCTs represent an important potential step in initiating the development of a cycling culture, but there is still a long way to go before cycling is more widely accepted as an alternative travel mode for many people. Other research has shown that increasing the cost of car travel (Pucher and Buehler, 2008), introducing improved cycling facilities and rights of way for cyclists (Parker et al., 2011; Pucher et al., 2010), and intensifying political pressure (Wachs, 1998) may all have a role in encouraging more cycling, and Wardman et al. (2007) suggest that cycling may have an appreciable impact on car use once a package of measures is implemented. For the bicycle to be regarded as a genuine sustainable alternative to the car in other locations, especially for shorter trips, it may be necessary to implement an integrated package of mutually reinforcing policies (such as a workplace parking policy), improved cycling facilities and infrastructure. The recent political pressure from the ‘Cities Fit for Cycling’ campaign has caught the attention of the UK Parliament (Burgess, 2012) and may contribute to creating a climate in which interventions of this kind might be put into practice.

## Figures and Tables

**Table 1:** Socio-demographic, transport and health-related characteristics of participants reporting work, shopping and leisure trips (%).

	Number of participants	Number of trips	Percentage of trips by car (cf. bicycle) for			
Work (*n* = 1395)	Work – less than 5 km (*n* = 534)	Shopping (*n* = 609)	Leisure (*n* = 622)
Total sample	1164	4105	46.7	13.3	67.5	61.5
Socio-demographic						
*Sex*						
Male	367	1242	34.8	10.5	63.5	48.8
Female	797	2852	53.0	15.1	68.7	65.6

*Age*						
<30 years	194	750	35.0	12.3	46.9	48.9
30–39 years	328	1156	41.9	9.2	76.3	65.4
40–49 years	303	1088	47.5	17.9	78.9	71.6
50–59 years	247	814	56.6	19.3	60.0	65.2
>60 years	88	278	56.6	9.1	85.7	41.7

*Child aged under 5*						
No	577	3464	48.1	14.4	66.0	60.7
Yes	167	630	38.5	7.9	79.5	68.6

*Child aged 5–15*						
No	552	3232	46.4	12.7	65.2	60.1
Yes	232	862	48.0	16.0	75.6	69.2

*Qualification*						
Degree	834	2919	40.6	10.8	61.5	54.6
‘A’ Level or equiv.	143	522	61.8	24.0	75.0	85.0
GCSE A–C or equiv.	106	365	69.0	33.3	88.9	88.5
Other	72	266	56.3	18.9	68.8	68.2

*Housing tenure*						
Rent	299	1073	28.4	12.0	49.0	45.3
Own	860	3009	52.3	13.6	75.0	67.9

*Urban vs. rural*						
Urban	993	3483	41.3	12.6	65.6	58.1
Rural	170	607	74.4	31.6	77.6	83.3
						
Transport						
*Driving licence*						
Yes	1049	3697	48.4	14.2	70.7	63.4
No	113	394	21.2	4.2	34.4	33.3

*Cars per adult*						
None	114	635	4.3	4.0	16.3	24.4
Less than one	112	1849	34.2	13.4	63.2	49.7
One or more	741	1548	72.3	26.1	88.9	85.4

*Commute distance*^a^						
Less than 3 km	146	543	8.7	8.7		
3–5 km	308	1109	14.9	14.9		
5–10 km	221	771	35.9			
10 km +	487	1661	84.6			

*Workplace parking*^a^						
Paid parking	351	1135	49.4	9.1		
Free parking	427	1551	55.4	24.3		
No parking	371	1362	33.8	7.3		

*Bicycle access*						
Yes	974	3490	40.1	12.6	62.9	59.5
No	182	585	100.0	100.0	100.0	100.0

*Walking for pleasure*						
0 min/week	226	802	39.7	11.0	52.7	53.0
<20 min/week	33	298	45.0	9.7	61.5	57.1
20–30 min/week	54	836	56.7	13.4	62.5	60.0
>30 min/week	332	381	51.2	2.8	74.8	55.4
						
Health						
*BMI*						
Normal/underweight	719	2548	40.5	12.0	63.3	56.8
Overweight	316	1112	53.2	12.9	75.2	73.4
Obese	110	365	68.2	26.3	80.8	86.7

PCS-8						
Mean (sd)			53.0(6)	52.4(8)		54.3(7)
(Bicycle)	53.7(7.8)	53.9(6.2)	(54.6(5))	(54.4(5))	(54.0(5))	(55.0(5))
*MCS-8*						
Mean (sd)			50.4(8)	51.4(8)	51.0(8)	49.8(8)
(Bicycle)	50.3(9.1)	50.6(8.0)	(51.4(7))	(51.4(7))	(51.2(7))	(51.5(8))

a Variable only available for work trips.

**Table 2:** Multivariable model of odds of choosing to travel by car for work trips.

	Univariable analysis	Model 1	Model 2	Model 3	Model 4
	P *R*^2^ 0.12	P *R*^2^ 0.45	P *R*^2^ 0.04	P *R*^2^ 0.52
	OR (95%CI)	OR (95%CI)	OR (95%CI)	OR (95%CI)	OR (95%CI)
Socio-demographic					
*Sex (reference: male)*					
Female	2.1 (1.5, 2.9)⁎⁎	2.1 (1.5, 3.0)⁎⁎			1.9 (0.9, 3.9)†

*Education (reference: degree)*					
A′ level or equiv	2.8 (1.2, 6.3)⁎	2.4 (1.5, 4.0)⁎⁎			4.7 (1.4, 15.8)⁎⁎
GCSE A–C or equiv	5.3 (1.7, 16.6)⁎⁎	2.3 (1.2, 4.3)⁎			2.0 (0.5, 7.1)
Other	2.0 (0.8, 5.0)	2.3 (1.3, 4.1)⁎⁎			1.6 (0.6, 5.0)

*Housing tenure (reference: rent)*					
Own	2.8 (1.9, 4.0)⁎⁎	2.7 (1.8, 4.2)⁎⁎			0.6 (0.3, 1.6)

Urban vs. rural (reference: urban)					
Rural	4.1 (2.7, 6.5)⁎⁎	4.2 (2.6, 6.8)⁎⁎			1.1 (0.5, 2.5)
					
Transport					
*Cars per adult (reference: none)*					
Less than one	11.7 (4.5, 30.6)⁎⁎		20.3 (3.3, 134.9)⁎⁎		56.6 (4.2, 746.3)⁎⁎
One or more	58.6 (22.2, 254.5)⁎⁎		47.5 (7.2, 316.1)⁎⁎		151.9 (11.5, 709.6)⁎⁎

*Commute distance (reference: less than 3 km)*					
3–5 km	2.9 (1.0, 8.9)†		2.6 (0.5, 13.6)		2.4 (0.5, 12.0)
5–10 km	5.6 (1.7, 18.6)⁎		7.4 (1.4, 37.6)⁎		8.1 (1.6, 38.6)⁎⁎
10 km+	33.3 (3.9, 284.7)⁎⁎		79.9 (15.6, 407.8)⁎⁎		104.1 (20.7, 524.0)⁎⁎

*Workplace parking (reference: paid parking)*					
Free parking	1.3 (0.9, 1.8)†		1.7 (0.9, 3.5)⁎		1.8 (0.9, 4.0)⁎
No parking	0.5 (0.4, 0.8)⁎⁎		0.9 (0.5, 1.7)		0.9 (0.4, 1.6)

*Walking for pleasure (reference: 0 min)*					
<20 min	0.9 (0.3, 3.3)		1.3 (0.5, 3.3)		1.1 (0.4, 3.5)
20–30 min	1.1 (0.4, 2.9)		2.0 (1.0, 3.8)⁎		1.5 (0.7, 3.6)
>30 min	0.3 (0.6, 1.5)		1.0 (0.5, 2.3)		0.8 (0.3, 1.7)
					
Health					
*BMI (reference normal/underweight)*					
Overweight	1.0 (0.5, 2.1)			1.7 (1.1, 2.33)⁎⁎	1.0 (0.5, 2.0)
Obese	3.3 (1.2, 8.9)⁎			3.1 (1.7, 5.4)⁎⁎	1.5 (0.6, 3.8)†

*PCS-8*					
	1.0 (0.9, 1.1)			1.0 (0.9, 1.1)†	1.0 (0.9, 1.1)

*MCS-8*					
	1.0 (0.9, 1.1)			1.0 (0.9, 1.0)⁎	1.0 (0.9, 1.0)

Model 1 – socio-demographic, Model 2 – transport, Model 3 – health, Model 4 – socio-demographic + transport + health.

P *R*^2^ – pseudo *R*-square value; OR – odds ratio; CI – confidence intervals.

† *p* < 0.10.

⁎ *p* < 0.05.

⁎⁎ *p* < 0.01.

**Table 3:** Multivariable analysis for odds of choosing to travel by car for short work trips – less than 5 km.

	Univariable analysis	Model 1	Model 2	Model 3	Model 4
		P *R*^2^ 0.06	P *R*^2^ 0.15	P *R*^2^ 0.05	P *R*^2^ 0.25
	OR (95%CI)	OR (95%CI)	OR (95%CI)	OR (95%CI)	OR (95%CI)
Socio-demographic					
*Education (reference: degree)*					
A′ level or equiv	2.6 (1.0, 6.5)⁎	2.6 (0.9, 7.2)†			4.2 (1.0, 18.2)⁎
GCSE ‘A–C’ or equiv	4.1 (1.1, 14.7)⁎	2.9 (0.7, 12.4)			6.1 (0.9, 40.6)⁎
Other	1.9 (0.7, 5.4)	2.0 (0.7, 5.7)			3.1 (0.9, 10.9)†

Transport					
*Car per adult (reference: none)*					
Less than one	2.6 (0.7, 9.2)		2.3 (0.5, 9.7)		2.3 (0.4, 12.8)
One or more	12.4 (3.7, 45.9)⁎⁎		9.7 (2.2, 42.8)⁎⁎		10.4 (2.1, 51.6)⁎⁎
					
*Workplace parking (reference: paid parking)*					
Free parking	3.2 (1.4, 7.6)⁎		3.1 (1.2, 7.6)⁎		4.3 (1.5, 12.2)⁎⁎
No parking	0.8 (0.3, 2.0)		0.9 (0.3, 2.7)		0.8 (0.2, 2.7)
Health					
					
*BMI (reference normal/underweight)*					
Overweight	1.1 (0.5, 2.5)			1.0 (0.4, 2.5)	0.7 (0.2, 2.1)
Obese	2.6 0.9, 7.6)			3.1 (0.9, 9.6)⁎	1.4 (0.4, 4.4)

Model 1 – socio-demographic, Model 2 – transport, Model 3 – health, Model 4 – socio-demographic + transport + health.

P *R*^2^ – pseudo *R*-square value; OR – odds ratio; CI – confidence intervals.

† *p* < 0.10.

⁎ *p* < 0.05.

⁎⁎ *p* < 0.01.

**Table 4:** Multivariable analysis for odds of choosing to travel by car for shopping trips.

	Univariable analysis	Model 1	Model 2	Model 3	Model 4
		P *R*^2^ 0.13	P *R*^2^ 0.23	P *R*^2^ 0.04	P *R*^2^ 0.36
	OR (95%CI)	OR (95%CI)	OR (95%CI)	OR (95%CI)	OR (95%CI)
Socio-demographic					
*Age (reference: <30 years)*					
30–39 years	3.7 (1.6, 8.4)⁎⁎	2.3 (0.8, 6.2)			1.5 (0.3, 9.1)
40–49 years	4.2 (1.8, 10.1)⁎⁎	2.5 (0.9, 6.7)†			5.4 (0.9, 31.5)†
50–59 years	1.7 (0.7, 4.0)	0.9 (0.3, 2.2)			2.9 (0.4, 19.4)
>60 years	6.8 (1.6, 28.3)⁎	3.4 (0.5, 16.3)			65.9 (4.9, 723.7)⁎⁎

*Education (reference: degree)*					
A′ level or equiv	1.9 (0.7, 4.9)	2.0 (0.8, 5.1)			14.8 (2.1, 118.5)⁎⁎
GCSE ‘A–C’ or equiv	5.0 (1.7, 14.7)⁎⁎	5.4 (1.8, 14.7)⁎⁎			17.3 (2.5, 144.0)⁎⁎
Other	1.4 (0.3, 5.9)	1.6 (0.3, 7.9)			2.2 (0.3, 16.1)

*Housing tenure (reference: rent)*					
Own	3.1 (1.6, 6.0)⁎⁎	2.7 (1.3, 5.6)⁎⁎			0.3 (0.1, 1.2)†
					
Transport					
*Car per adult (reference: none)*					
Less than one	8.8 (2.8, 27.7)⁎		6.8 (1.8, 25.1)⁎⁎		14.5 (1.8, 117.3)⁎⁎
One or more	41.0 (11.7, 143.2) ⁎		35.3 (8.2, 153.2)⁎⁎		103.3 (10.8, 778.7)⁎⁎

*Walking for pleasure (reference: 0 min)*					
<20 min	1.5 (0.4, 5.4)		1.1 (0.3, 3.8)		0.4 (0.1, 2.5)
20–30 min	3.1 (1.2, 7.8)⁎		2.7 (0.9, 8.6)†		2.4 (0.8, 7.4)
>30 min	2.0 (0.7, 5.9)		1.0 (0.3, 3.2)		1.6 (0.4, 5.7)

Model 1 – socio-demographic, Model 2 – transport, Model 3 – health, Model 4 – socio-demographic + transport + health.

P *R*^2^ – pseudo *R*-square value; OR – odds ratio; CI – confidence intervals.

† *p* < 0.10.

⁎ *p* < 0.05.

⁎⁎ *p* < 0.0.

**Table 5:** Multivariable analysis for odds of choosing to travel by car for leisure trips.

	Univariable analysis	Model 1	Model 2	Model 3	Model 4
		P *R*^2^ 0.13	P *R*^2^ 0.17	P *R*^2^ 0.04	P *R*^2^ 0.27
	OR (95%CI)	OR (95%CI)	OR (95%CI)	OR (95%CI)	OR (95%CI)
Socio-demographic					
*Sex (reference: male)*					
Female	2.0 (1.1, 4.0)⁎	2.2 (1.0, 4.7)⁎			2.1 (0.8, 5.4)

Education (reference: degree)					
A′ level or equiv	4.7 (1.4, 15.6)⁎	4.2 (1.3, 13.2)⁎⁎			3.8 (1.1, 14.6)⁎
GCSE ‘A–C’ or equiv	6.4 (1.3, 31.8)⁎	8.1 (1.3, 50.9)⁎			14.8 (2.3, 95.9)⁎⁎
Other	1.5 (0.4, 5.3)	1.3 (0.4, 4.6)			0.7 (0.2, 2.9)

*Urban vs. rural (reference: urban)*					
Rural	3.6 (1.3, 9.9)⁎	3.4 (1.1, 9.9)⁎			1.6 (0.5, 4.9)
					
Transport					
*Car per adult (reference: none)*					
Less than one	3.1 (1.1, 8.2)⁎		2.7 (1.0, 7.5)⁎		2.2 (0.6, 8.1)
One or more	18.1 (6.2, 52.7)⁎⁎		16.2 (5.4, 48.5)⁎⁎		11.2 (2.8, 44.8)⁎⁎
					
Health					
*BMI (reference normal/underweight)*					
Overweight	2.8 (0.3, 25.1)			2.0 (0.9, 4.6)	1.8 (0.6, 8.1)
Obese	6.5 (0.6, 76.5)			4.6 (1.2, 18.5)⁎	3.8 (0.8, 17.3)†

Model 1 – socio-demographic, Model 2 – transport, Model 3 – health, Model 4 – socio-demographic + transport + health.

P *R*^2^ – pseudo *R*-square value; OR – odds ratio; CI – confidence intervals.

† *p* < 0.10.

⁎ *p* < 0.05.

⁎⁎ *p* < 0.00.
